# Supplementary material for: Association between Muscle Fatigability, Self-Perceived Fatigue and C-Reactive Protein at Admission in Hospitalized Geriatric Patients
Source: Int J Environ Res Public Health. 2023 Aug 16;20(16):6582. doi: 10.3390/ijerph20166582 (PMC10454850; doi:10.3390/ijerph20166582)
Supplement: Supplementary file 1 [file ijerph-20-06582-s001.zip › ijerph-2377626-supplementary.pdf]

Supplementary Materials

Table S1. Baseline characteristics compared between participants with missing data and participants with complete data for confounding variables.

	Complete data (n=86)	Missing data* (n=18)	p-value**
**Age (yrs, mean ±SD)**	83.1 ± 7.5	84.3 ± 7.3	0.57
**Weight (kg, mean ±SD)**	72.5 ± 16.1	69.2 ± 15.4	0.43
**CSF (mean ±SD)**	5.5 ± 1.4	5.6 ± 1.2	0.72
**BRS (mean ±SD)**	3.1 ± 0.8	n.a.	
**Grip Strength (kPa, mean ±SD)**	40.5 ± 16.9	39.7 ± 20.3	0.86
**Mortality during admission (% deceased)**	23.9	44.4	0.14
**No chronic conditions (median [IQR])**	4.0 [2.0–5.0]	3.0 [1.3–4.0]	0.08
**Length of stay (days, median [IQR])**	7.0 [4.0–11.0]	4.5 [4.0–7.8]	0.10
**Fatigue resistance (s, median [IQR])**	24.0 [16.0–40.0]	29.5 [21.5–36.8]	0.34
**Grip Work (median [IQR])**	667.5 [334.7–1170.0]	682.5 [501.0–1510.0]	0.55
**SPF (median [IQR])**	6.0 [5.0–8.0]	5.0 [2.3–7.5]	0.07
**CPV (median [IQR])**	1.6 [0.8–3.4]	3.4 [1.1–7.7]	0.07
**CRP (mg/L, median [IQR])**	19 [4.0–54.0]	59 [14.8–104.8]	0.04
**Time between PCV and CRP (days, median [IQR])**	1.5 [1.0–2.0]	1.0 [0.0–1.0]	<0.001

BRS, brief resilience scale; CFS, clinical frailty scale; CPV, capacity to perceived vitality ratio; CRP, C-reactive protein; IQR, interquartile range; SD, standard deviation.

* The only confounder with missing data was the brief resilience scale (BRS).

** Differences were examined using the t-test for normally distributed continuous variables, the sum rank test for not-normally distributed continuous variables and the chi-squared test for categorical variables.
